# GND-PCA-Based Statistical Modeling of Diaphragm Motion Extracted from 4D MRI

**DOI:** 10.1155/2013/482941

**Published:** 2013-05-26

**Authors:** Windra Swastika, Yoshitada Masuda, Rui Xu, Shoji Kido, Yen-Wei Chen, Hideaki Haneishi

**Affiliations:** ^1^Graduate School of Engineering, Chiba University, Chiba 263-8522, Japan; ^2^Chiba University Hospital, Chiba 260-8677, Japan; ^3^Graduate School of Medicine, Yamaguchi University, Yamaguchi 755-8505, Japan; ^4^Graduate School of Science and Engineering, Ritsumeikan University, Shiga 525-8577, Japan; ^5^Research Center for Frontier Medical Engineering Chiba University, Chiba 263-8522, Japan

## Abstract

We analyzed a statistical model of diaphragm motion using regular principal component analysis (PCA) and generalized N-dimensional PCA (GND-PCA). First, we generate 4D MRI of respiratory motion from 2D MRI using an intersection profile method. We then extract semiautomatically the diaphragm boundary from the 4D-MRI to get subject-specific diaphragm motion. In order to build a general statistical model of diaphragm motion, we normalize the diaphragm motion in time and spatial domains and evaluate the diaphragm motion model of 10 healthy subjects by applying regular PCA and GND-PCA. We also validate the results using the leave-one-out method. The results show that the first three principal components of regular PCA contain more than 98% of the total variation of diaphragm motion. However, validation using leave-one-out method gives up to 5.0 mm mean of error for right diaphragm motion and 3.8 mm mean of error for left diaphragm motion. Model analysis using GND-PCA provides about 1 mm margin of error and is able to reconstruct the diaphragm model by fewer samples.

## 1. Introduction

4D-MRI is an advanced imaging technique that reconstructs a 3D MRI with time series from a set of time sequential images of 2D MRI. For respiratory motion, the use of 4D MRI has an important role in many clinical applications such as lung cancer radiotherapy planning, examining pulmonary diseases, and analyzing diaphragm motion. However, current MRI is unable to acquire 4D MRI directly. Therefore, in the recent years some methods have been proposed to reconstruct 4D MRI of respiratory organs based on the sequential 2D MRI [1–3]. 

von Siebenthal et al. [1] proposed a method to obtain 4D image using internal respiratory gating and reconstructed it by retrospective sorting of dynamic 2D MR images. It showed the detailed deformation of an organ during free breathing. Tokuda also proposed an adaptive imaging method to acquire a series of 3D MR images of respiratory organs as the extension of respiratory gating [2]. Our previous study successfully achieved 4D MR imaging of organs with respiratory motion using a method called intersection profile method [3]. In this method, we reconstructed 4D MRI of respiratory organ from time sequential images of 2D MRI under natural respiration. We not only successfully visualized 4D MRI of respiratory organ, but also proposed to construct diaphragmatic function map that can be used to evaluate diaphragm motion quantitatively. Previous related works on 4D respiratory motion modeling include [4–8]. 

Recent statistical model of respiratory motion was proposed by Li et al. [9]. It was a statistical model of lung based on principal component analysis (PCA) and applied to clinical data. The lung motion model, however, was based on two types of respiratory phantoms and cosine function which will only be idealistic for phantom motion. Extraction and statistical modeling of lung motion field were also demonstrated in [10]. The experiment extracted motion fields from a 4D-CT data set and built a motion model for both intra- and intersubject. Although it focused on the lung motion, the results showed that the use of diaphragm as a stimulator to drive the motion model could reduce the prediction error. Simultaneous registration of all dynamic MR images and modeling processing were performed in [11] for the purpose to improve the accuracy of motion estimation. However, this approach may only be feasible for simple rigid or affine motion model. Applying this model to organs that have complex or nonrigid motion will significantly increase the number of parameter and consequently execution time also becomes much larger.

The statistical modeling was focusing on how to model respiratory motion based on lung motion [9–11] or internal liver motion [12]. In this paper, instead of extracting lung to obtain respiratory motion, we focus on extracting diaphragm motion from 4D-MRI and analyzing it using PCA. As one of the major determinants in respiratory motion, diaphragm has greater superior-interior translation compared with other respiratory organs such as lung or liver. Thus, by modeling and analyzing diaphragm motion, the variability of respiratory motion can be clearly visualized. As mentioned in [13], GND-PCA method can construct MR T1-weighted brain volumes and CT lung volumes using fewer training samples compared with regular PCA. Hence, we also interested to analyze the efficacy of GND-PCA compared with regular PCA in modeling the diaphragm motion.

To the best of our knowledge, this is the first study of modeling and analyzing diaphragm motion extracted from 4D MRI.

## 2. Materials and Methods

The process of constructing and analyzing the diaphragm motion model consists of four parts. The first is diaphragm segmentation and motion tracking. We will briefly review the methodology we used to create 3D model of diaphragm shape. Second is data normalization. We will cover how to normalize the data obtained from the previous step. This step is primarily important to generalize the data from different subjects. Third is model analysis using PCA and GND-PCA. Basic theory of PCA and GND-PCA will be described. Last is data evaluation, in which we will explain how to validate the constructed model.

### 2.1. 3D Diaphragm Segmentation and Motion Tracking

**Figure 1:**
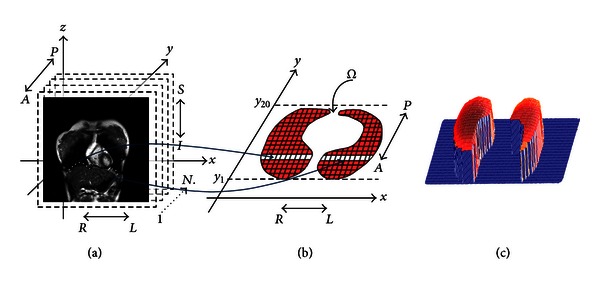
Coordinate definition of 4D MRI and diaphragm extraction. (a) Manually selected several points (white dots) of one data slice to extract diaphragm surface, (b) complete extraction of diaphragm surface from *y*_1_ to *y*_20_, and (c) representation of diaphragm area.

Right-handed Cartesian coordinate system is used to cover the whole diaphragm area. A number of MRI data slices, size of 256 × 256 pixels in coronal view are set along the *y*-axis (Figure 1(a)). Each data slice position is denoted by *y*_*i*_ where *i* = 1,…, *S*. To assess the diaphragm motion, we also use *T* time-sequential images for each data slice of *y*_*i*_. We denote one data slice as *f*_data_(*x*, *y*_*i*_, *z*, *t*), where *t* = 1,2,…, *T*. A first shape of diaphragm is obtained from *f*_data_(*x*, *y*_*i*_, *z*, *t*) for *i* = 1,2,…, *S* and *t* = 1. The diaphragm shape is determined as follows. Several points are selected in each data slice (*y*_*i*_) that represents diaphragm boundary shown as white dots in Figure 1(a). The number of points varies from 10 to 15 depending on the curve of diaphragm boundary. Generally, more points are required if the diaphragm boundary has a rounded or curvature shape. The points are then connected by spline interpolation. By conducting this operation for all  *y*_*i*_, the area of diaphragm in *xy* plane is defined as shown in Figure 1(b). We denote this shape as *Ω*. Note that we ignore the area below the heart because it is strongly affected by heart beat and apart from respiratory motion. The entry of this matrix represents *z* value of diaphragm surface at (*x*, *y*). The 3D representation of diaphragm surface is shown in Figure 1(c). In this step, *S* × 256 matrix for one whole diaphragm area is generated, where *S* is the number of slice. Depending on the acquisition process, *S* will vary between 16 and 24.

Once the 3D shape on diaphragm area *Ω* is obtained for *t* = 1 (as shown in Figure 1(b)), the 3D shape of diaphragm over the area of Ω is tracked in the next frame. In order to do so, a profile of *f*_data_(*x*, *y*_*i*_, *z*, *t*) along *Z* at position (*x*, *y*) over Ω is compared with a profile of *f*_data_(*x*, *y*_*i*_, *z*, *t* − 1) at the same position (*x*, *y*_*i*_), and is found a value of displacement of diaphragm along *z*-axis by using normalized cross-correlation. 

**Figure 2:**
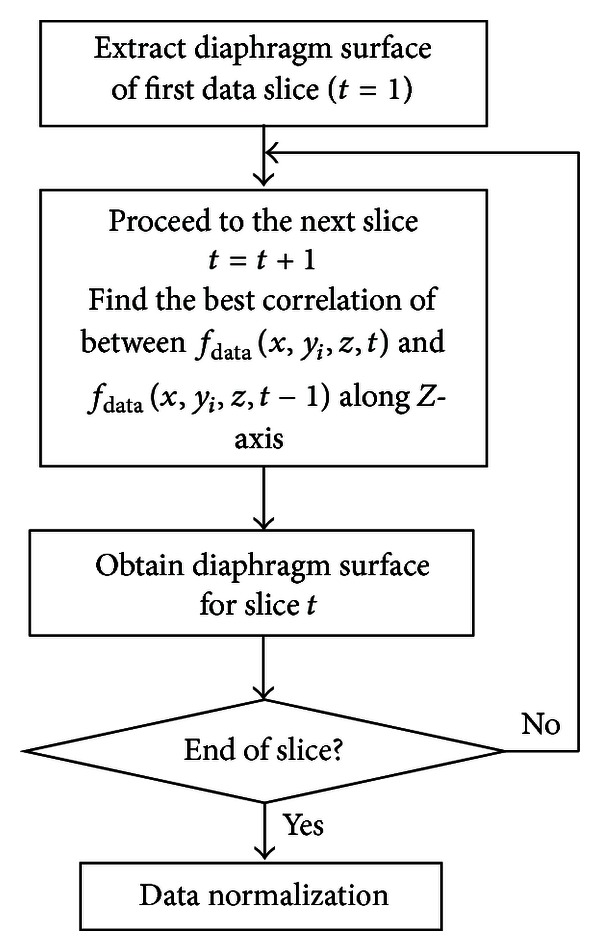
Flow diagram of diaphragm motion tracking method.

Figure 2 summarizes the flow diagram of diaphragm motion tracking method. Complete reference regarding this motion tracking method can be found in [3].

### 2.2. Data Normalization

The acquisition of diaphragm motion based on the previously explained method cannot be generalized for all diaphragms due to the wide range of variability of diaphragm shape and size among the subjects. Hence, the acquired data need to be normalized. The normalization process takes four steps. 

First, we divide the diaphragm area into two parts: right and left diaphragm areas. 

Second, to represent a detailed and unique diaphragm region, we calculate gradient edges of all diaphragm shapes and choose one that has the highest gradient edges as reference image. Affine registration is then performed for all diaphragm shapes to ensure the same location and size of all diaphragm shape before the analysis is performed.

Third, for each diaphragm area, we set the top left and bottom right coordinate to limit the diaphragm area into a rectangular shape. The distance of new top left position is 1/10 of the diaphragm area width and so is the new bottom right position. Figure 2 shows how to set new region of diaphragm area. Selecting the region of diaphragm area ensures that the analysis is only done in the main part of the diaphragm area and ignores the area that has small movement. This also maintains the correspondence on different subjects since the top and bottom areas will constantly represent same anterior and posterior positions of all subjects.

The last step of normalization process consists of two parts, temporal and spatial normalization. Temporal normalization makes all subjects have the same number of frames, while spatial normalization only normalizes the size of diaphragm area.

Let *Z*(*x*, *y*, *t*) denotes the *z* value of diaphragm surface (or target image) at (*x*, *y*) position and *t*th frame (1,2,…, *T*). After temporal normalization, *Z*(*x*, *y*, *t*) can be denoted as *Z*(*x*, *y*, t*), where t* ranges from 1 to 20. The following operator is used to define t*:

(1) $$\begin{array}{c} t^{*}=\left\lfloor \frac{20-1}{T-1}\left(t-1\right)+1\right\rfloor . \end{array}$$

Here the operator ⌊ ⌋ represents ceiling function which returns a decimal number to its smallest integer.

**Figure 3:**
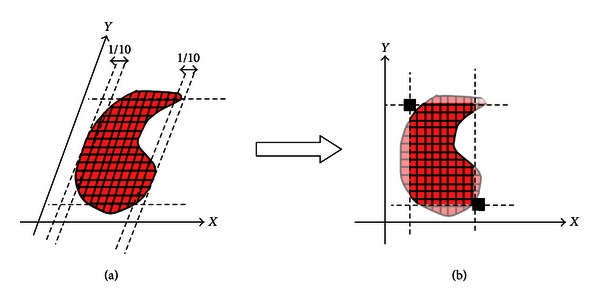
Set the top left and bottom right coordinate to limit the diaphragm area.

Due to the fact that the coordinate position and the size of rectangular area shown in Figure 3(b) vary among the subjects, the last part in the normalization process is to fix the rectangular area for both *y* and *x* axes. The purpose is that all the data will have the same size and position. The size of the reference image is represented by *W*_ref_ · *H*_ref_. In our study, we used 60 × 100 pixels for *W*_ref_ · *H*_ref_. Actual normalization process is described as follows.

(1) *Normalization of y-Axis*. To normalize the diaphragm area into *Y* axis, the origin image is scaled and fixed to the reference image. The following operator is used to scale diaphragm area in *y*-axis,
  (2) $$\begin{array}{c} Z^{\prime }\left(x,y,t\right)=Z\left(x,\left\lceil \frac{H_{\text{target}}}{H_{\text{ref}}}y\right\rceil ,t^{*}\right), \end{array}$$
  where *H*_target_ is height of the image target and *H*_ref_ is the height of the reference image. Operator ⌈*x*⌉ represents ceiling function which rounds up the decimals into an integer. This scaling process is done for all *x* and *y*. The results of *y*-axis normalization is called intermediate image.
(2) *Normalization of x-Axis*. The width of the reference image is also fixed by horizontal scaling. The operator used to scale diaphragm area in *x*-axis is written as
  (3) Z′′(x,y,t) =Z′(⌈Wtarget(n)−Wtarget(1)Wrefx+Wtarget(1)⌉,y,t∗),
  where *W*_target_(*n*) and *W*_target_(1) are the last and first nonzero positions in the current *y*-axis and *W*_ref_ is the width of the reference image. This horizontal scaling is done for all *x*, *y*. The result of *x*-axis normalization is a final image with the same width and height of the reference image.

Since there are both right and left diaphragm areas, scaling the area using the reference image is done for both diaphragm areas. This process is repeated for each data frame obtained from the previous algorithm starting from the first time-sequential image to the last one (t* = 1 ⋯ 20). 

The matrix dimension of diaphragm motion after normalization is *W*_ref_ × *H*_ref_ × 20 or equals to 60 × 100 × 20 (spatial size of reference image ×20 frames) for each side of diaphragm. To ensure that the diaphragm motion is represented as a whole diaphragm and keeping the shape variance, we merge the right and left sides of diaphragm into a matrix. The final matrix dimension after the merging is 60 × 200 × 20. Considering the data as high-dimensional data, linear statistical analysis is possible to be carried out by applying principal component analysis (PCA). It reduces the data set and reveals the hidden pattern as maintaining the majority of the variation in the original data. 

The upper part of Figure 3 shows the spatial normalization process of certain frame. After modeling using PCA, we reverse the image into the original diaphragm shape. Firstly, we create a mask based on the original diaphragm shape. Using this mask, the modeled image is then resized and reshaped to the original diaphragm shape. The bottom part of Figure 3 shows the reversing process from a frame modeled by PCA to a diaphragm shape image.

### 2.3. PCA and GND-PCA Diaphragm Motion Model

Generally, PCA is a statistical method to transform a set of correlated variables into a smaller number of uncorrelated variables or principal components (PCs). The PCs are sorted in a descending order of importance. The purpose of PCA is that the first few PCs are able to explain large proportion of the variation in the original variables, and only those PCs are retained for further analysis.

The following paragraphs describe how PCA is used to analyze diaphragm motion. Let **z** be a vector of *z* value of the spatiotemporally normalized for both right and left diaphragm. Vector **z** can be expressed as 1D array:

(4) $$\begin{array}{c} z={\left[z_{1},z_{2},z_{3},\ldots ,z_{N}\right]}^{T}, \end{array}$$

where

(5) $$\begin{array}{c} z_{i}=Z\left(x,y,t\right) \end{array}$$

and *i* is the index obtained by the following equation:

(6) $$\begin{array}{c} i=\left(t-1\right)H_{\text{ref}}W_{\text{ref}}+\left(y-1\right)H_{\text{ref}}+x. \end{array}$$

For *m* subjects, we denote **z** ^(*j*)^  (*j* = 1,2, 3,…, *m*) as a diaphragm motion data set from *j*th subject.

Principal components are the eigenvectors with its corresponding eigenvalues of covariance matrix of **z**. The sorted eigenvectors by decreasing order of its corresponding eigenvalues is the most optimal with respect to information loss.

Another method to build a statistical method is GND-PCA. It is a method to model a series of multidimensional array proposed by McQuaid et al. [14]. Instead of using one long vector to represent a motion model, GND-PCA uses a tensor to represent a shape or motion model. The tensor itself is a multidimensional array whose order is the number of dimensions, also known as ways or modes. We will give a brief explanation of GND-PCA. More details about GND-PCA can be read in [14].

An *N*th-order tensor, denoted by *𝒜*, where *𝒜* ∈ *R* ^*I*_1_×*I*_2_×*I*_3_×⋯×*I*_*n*_^ and *R* ^*n*^ denotes the set of all vectors with *n* real components. In tensor point of view, a vector and a matrix are a tensor of order one and order two, respectively. One diaphragm motion can be considered as third-order tensor *ℳ*, where *ℳ* ∈ *R* ^*I*_1_×*I*_2_×*I*_3_^ (*I*_1_ × *I*_2_ is the spatial dimension of the diaphragm in each frame and *I*_3_ is the number of frame).

Here, let *ℳ*_*i*_  (*i* = 1,2, 3,…, *m*) denote *m* samples of third-order tensor that represents diaphragm motion from *m* subjects. A series of lower rank tensors *ℳ*_i_* ∈ *R* ^*J*_1_×*J*_2_×*J*_3_^ is defined as the most accurate approximation of original tensors *ℳ*_*i*_, where *J*_1_ < *I*_1_, *J*_2_ < *I*_2_ and *J*_3_ < *I*_3_. To obtain *ℳ*_i_*, we decompose the tensors into smaller core tensors, and their corresponding orthogonal mode matrices are shown by:

(7) ℳi∗=𝒞i×Y1×X2×T3.

**Figure 4:**
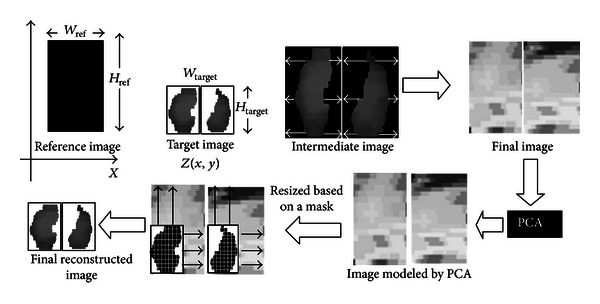
Diagram of spatial normalization and its reconstruction process to obtain diaphragm original shape.

The product _*n*_ **X** denotes the *n*-mode product between the tensor and the mode matrices [14].  Figure 4 shows the illustration of 3rd-order tensor decomposition of diaphragm motion model.

The orthogonal mode matrices capture the variation along the spatial (**Y** ∈ *R* ^*I*_1_×*J*_1_^ and **X** ∈ *R* ^*I*_2_×*J*_2_^) and time (**T** ∈ *R* ^*I*_3_×*J*_3_^) dimension. The core tensors (*𝒞*_*i*_ ∈ *R* ^*J*_1_×*J*_2_×*J*_3_^) control the interaction between mode matrices and can be seen as the compressed version of the original tensor. The mode matrices can be obtained by solving the following equation:

(8) min⁡||ℳi  −  ℳi∗||=min⁡||ℳi−𝒞i×Y1×X2×T3||.

### 2.4. Evaluation Methods

In this study, we evaluate the performance of the diaphragm motion model by calculating the mean and maximum errors of the constructed model. Leave-one-out method is used for this evaluation [15]. 

The error of approximated model from each subject can be obtained by simply subtracting each of the elements of constructed model from the original shape and getting the absolute value. The error of right and left diaphragm shapes of *j*th subject can be mathematically written as

(9) $$\begin{array}{c} e^{\left(j\right)}\left(x,y,t\right)=\left|{\hat{z}}^{j}\left(x,y,t\right)-z^{\left(j\right)}\left(x,y,t\right)\right|. \end{array}$$

Here, we redefined the shape of normalized diaphragm by *z* ^(*j*)^(*x*, *y*, *t*). We also represent the estimate by the statistical model by ${\hat{z}}^{\left(j\right)}\left(x,y,t\right)$.

Based on this definition of error, some kinds of mean or maximum error can be expressed. For instance, mean error of each subject is given by

(10) $$\begin{array}{c} e_{\text{mean}}^{\left(j\right)}=\frac{1}{n^{\left(j\right)}T}\sum_{t=1}^{T}\;\;\sum_{x,y\in \Omega^{\left(j\right)}}e_{k}^{\left(j\right)}\left(x,y,t\right), \end{array}$$

where *n* ^(*j*)^ is the number of nonzero values in the diaphragm area *Ω* ^(*j*)^ of *j*th subject. Intersubject average of *e*_*k*,mean_^(*j*)^ is given by:

(11) $$\begin{array}{c} e_{\text{mean}}=\frac{1}{m}\sum_{j=1}^{m}e_{\text{mean}}^{\left(j\right)}. \end{array}$$

The maximum error for *j*th subject is given by

(12) $$\begin{array}{c} e_{\max}^{\left(j\right)}=\max\left(e^{\left(j\right)}\left(x,y,t\right)\right). \end{array}$$

We can also calculate the intersubject average of maximum error by

(13) $$\begin{array}{c} e_{\max}=\frac{1}{m}\sum_{j=1}^{m}e_{\max}^{\left(j\right)}. \end{array}$$

Another evaluation method we used is frame-by-frame error calculation. Frame-by-frame error is important to analyze which respiratory phase gives the largest or smallest error. Frame-by-frame mean error of each subject is given by

(14) $$\begin{array}{c} e^{\left(j\right)}\left(t\right)=\frac{1}{n^{\left(j\right)}}\sum_{x,y}e^{\left(j\right)}\left(x,y,t\right). \end{array}$$

Intersubject average of frame-by-frame error is given by

(15) $$\begin{array}{c} e\left(t\right)=\frac{1}{m}\sum_{j=1}^{m}e^{\left(j\right)}\left(t\right). \end{array}$$

## 3. Experimental Results

Ten healthy subjects within the age ranging from 23 to 46 participated in this study. For diaphragm motion studies, MR images are particularly preferred than CT images since MR images provide high soft tissue contrast to produce detailed respiratory organs. The high contrast of MR images will be useful during the manual diaphragm boundary segmentation process.

In this study, MR Images were acquired using 1.5 T INTERA ACHIVA nova-dual (Philips Medical Systems) whole-body scanner with a 16ch SENSE TORSO XL Coil. A 2D balanced FFE sequence was used. The imaging parameters are as follows: SENSE factor: 2.2, flip angle: 45°, TR: 2.2 ms, TE: 0.9 ms, FOV: 384 mm, in-plane resolution 256 × 256 pixels and 1.5 × 1.5 mm^2^, slice thickness: 7.5 mm, slice gap = 6.0 mm, and scan time: 150 ms/frame and 400 frame/slice. Normal breathing was instructed for all subjects during the acquisition process. This image acquisition experiment was conducted under an approval of Ethical Review Board of Chiba University.

The software used for PCA is MATLAB 7.10 and running on PC with Intel Core 2 Quad, 2.66 GHz, 16 GB RAM.

### 3.1. PCA and GND-PCA Model Output

The contribution ratio and cumulative up to three principal components of right and left diaphragm motion are listed in Table 1. The percentage of variance of first principal component is 97.4% and 99.2% for the first three principal components. 

**Figure 5:**
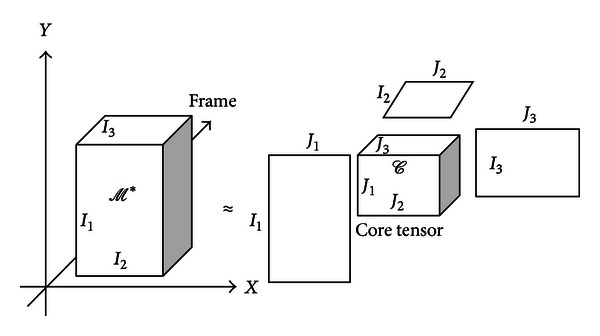
Decomposition of 3rd-order tensor into one core tensor and three mode matrices.

**Figure 6:**
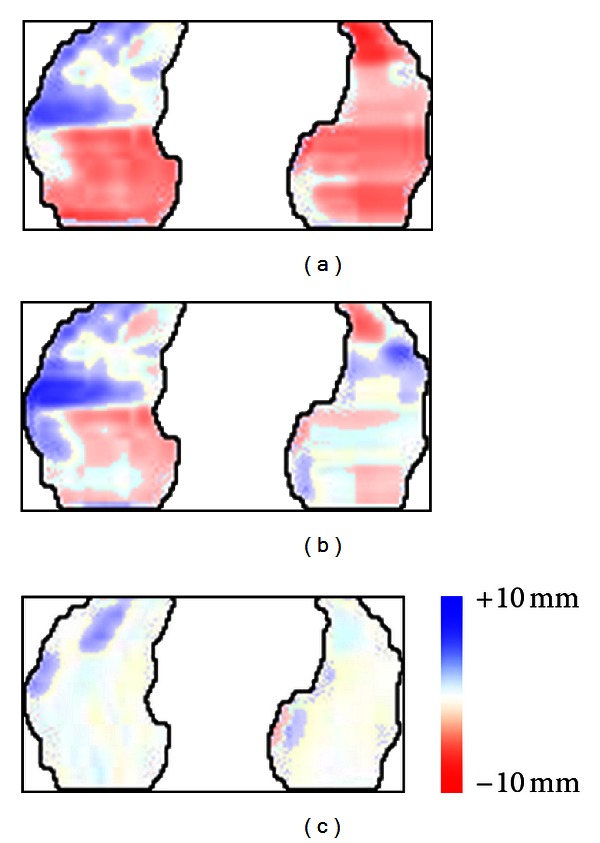
Regular PCA Error position mapping of one frame using (a) first PC, (b) first two PCs, and (c) first three PCs.

Mapping the error of *z* coordinates in the constructed model using different number of PCs will be useful for further analysis. Figures 5 and 6 illustrate color mapping of the error in the first frame of the first subject given by

(16) $$\begin{array}{c} e^{\left(1\right)}\left(x,y,1\right)={\hat{z}}^{\left(1\right)}\left(x,y,1\right)-z^{\left(1\right)}\left(x,y,1\right). \end{array}$$

Note that the error is not an absolute value as expressed in (9).

The white area represents the exact approximation, red and blue areas indicate that estimated *z* coordinates are higher and lower than the actual position, respectively.

Figure 6 is the case when first one, first two, and first three PCs are used in regular PCA, respectively. As shown in Figure 6(c), the red and blue areas are decreasing. This indicates that the model well approximated the actual shape when the first three PCs were used.

**Figure 7:**
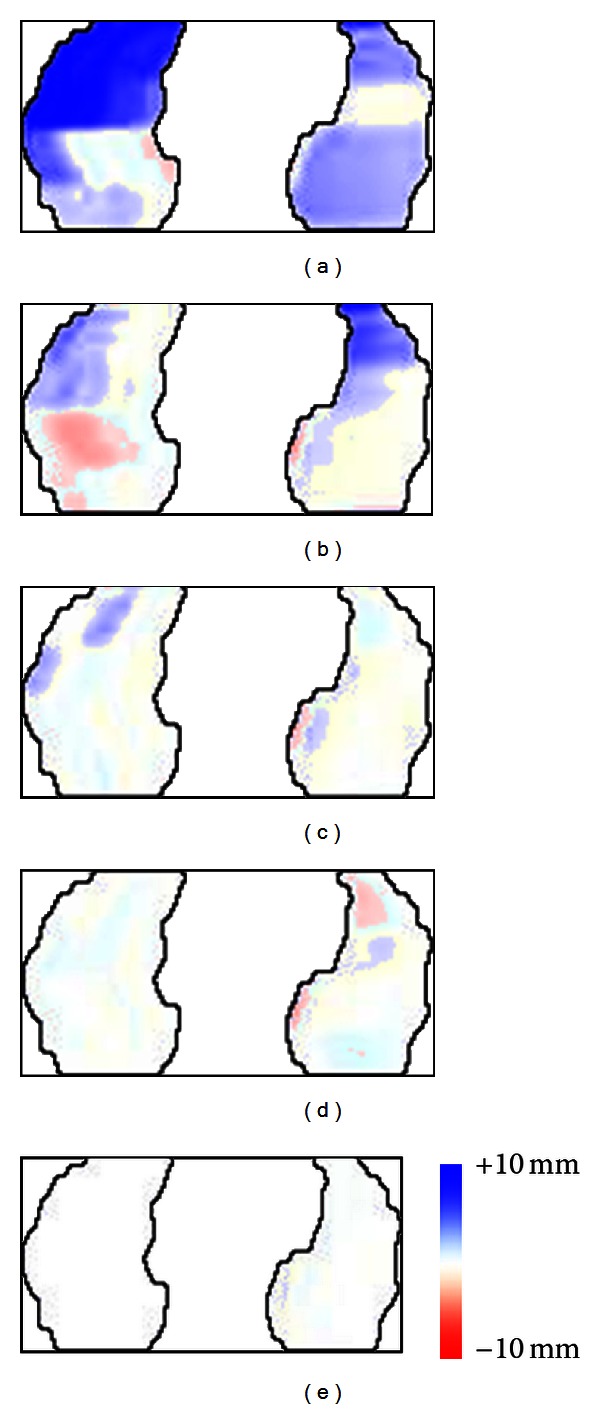
GND-PCA error position mapping of one frame using (a) 4 × 2 × 1, (b) 8 × 4 × 2, (c) 16 × 8 × 4, (d) 32 × 16 × 8, and (e) 64 × 32 × 16, core tensors.

The similar results are also shown by GND-PCA construction as illustrated in Figure 7. The error color mappings were obtained by reconstructing the model with 4 × 2 × 1, 8 × 4 × 2, 16 × 8 × 4, 32 × 16 × 8, and 64 × 32 × 16 core tensors respectively and subtracting them from the original shape of diaphragm. The last three core tensors showed that the red and blue colors on the diaphragm area almost disappeared, which means the constructed models are very similar to the original shape.

In term of number of coefficients required to construct the model, regular PCA outperformed the GND-PCA.  Table 2 shows the comparison of the number of coefficients required to construct diaphragm motion model between regular PCA and GND-PCA.

### 3.2. Leave-One-Out Method Validation

We omitted one subject as a testing subject and constructed the diaphragm motion model using training data from the remaining nine subjects. This model was then applied to the testing subject. The mean error of the testing subject was calculated using (10). The whole procedure is repeated till each of ten subjects has become testing subject once. 

**Figure 8:**
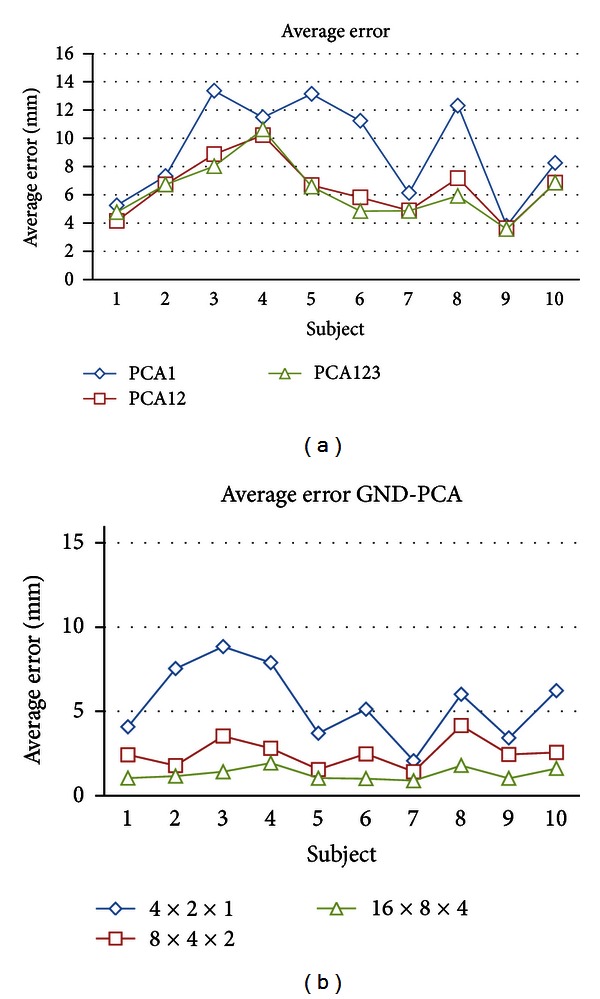
The mean error of the model by (a) regular PCA from and (b) GND-PCA. The measurement unit is in mm.

Figure 8(a) shows the mean error of each subject in case of model using regular PCA and GND-PCA, respectively. For regular PCA model, the mean error ranges are 3.8–13.4 mm for first PC, 3.6–10.2 mm for first two PCs, and 3.5–10.6 mm for first three PCs. Although more than 98% variability of the diaphragm motion can be covered by the first three PCs as shown in Table 1, the validation using leave-one-out method showed that intersubjecting the average of mean error of the model given by (11) is more than 4 mm.

Contrary to the regular PCA, the error mean of model by GND-PCA as shown in Figure 8(b) is much smaller. The mean error ranges are 1.4–9.0 mm for 4 × 2 ×  1 core tensor, 1.4–4.0 mm for 8 × 4 × 2 core tensor, and 0.8–2.1 mm for 16 × 8 ×  4 core tensor.

**Figure 9:**
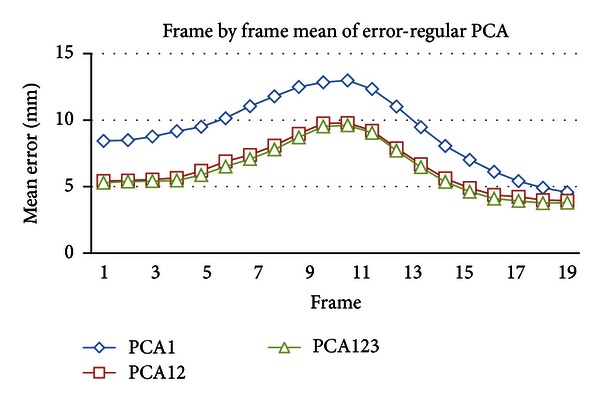
Regular PCA mean of error frame-by-frame.

Figure 9 showed frame-by-frame mean error *e*(*t*) of the model by regular PCA. As shown in this figure, 18–20th frames indicate low mean error (about 3.9 mm on average) and 9–11th frames indicate high mean error (about 9.0 mm on average). It is probably caused by the smaller variability in the 18–20th frames and higher variability in 9–12th frames.

**Figure 10:**
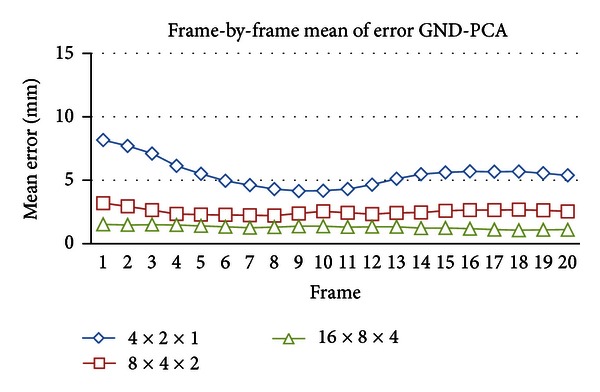
GND-PCA mean of error frame-by-frame.

Different results were obtained using GND-PCA as shown in Figure 10. Since GND-PCA can capture both spatial and time variability; there were no large differences of mean error among the frames. For instance, the standard deviation of mean error from 16 × 8 × 4 core tensors is 0.37 mm, while for three principal components of regular PCA the mean error is 2.2 mm.

Tables 2 and 3 summarize the average of mean error *e*_mean_ and average of maximum error *e*_max⁡_. The results of GND-PCA showed consistent reconstruction with smaller error compared with the model constructed using regular PCA.

## 4. Discussion

In this paper, we described how to build a statistical model of diaphragm motion using PCA and GND-PCA. The model was obtained from 4D MRI that reconstructed from time sequential images of thoracic 2D MRI of ten healthy subjects. The modeling process involves manual segmentation of diaphragm boundary, automatic motion tracking based on the intersection profile method [3], constructing region of interest for right and left areas of diaphragm, and normalization of diaphragm shape. 

The developed model using regular PCA can accurately describe more than 98% of the total variation by including the first three PCs. This indicates that most of the diaphragm motion variability is adequately described using a few number of parameters. Consequently, the description and motion of the diaphragm are greatly simplified by this model.

Leave-one-out validation was employed to evaluate the performance of the model. As shown in Table 2, the results of regular PCA illustrated that mean error position of both sides of diaphragm was more than 6.0 mm, which considered as significant error. 

To build a better statistical modeling, we applied GND-PCA [14]. Differing from regular PCA, GND-PCA is not necessarily unfolding the diaphragm motion model into one long vector. Instead, it decomposes the model into a core tensor and several mode matrices for dimensionality reduction. The mode matrices can represent the principal axes of variation. Several numbers of core tensors are chosen to construct the motion model. The smallest size of core tensor is 4 × 2 × 1 which is able to construct the motion model under leave-one-out validation with mean error of 5.5 mm. Among the three sizes of core tensor (4 × 2 × 1, 8 × 4 × 2, and 16 × 8 × 4); the best model construction is achieved by 16 × 8 × 4 core tensor which gives mean error of 1.3 mm. The maximum error is also significantly reduced to 5.6 mm. Compared with regular PCA using first three PCs, the results of GND-PCA showed significant improvement to the motion model. Mean error obtained from frame-by-frame analysis shown in Figure 10 also confirmed that GND-PCA is able to capture the motion variability of the diaphragm. One of the major drawbacks of GND-PCA is that it requires more coefficients to construct the model compared with regular PCA.

 There are some considerations regarding the diaphragm motion model in this study. The first is the resolution of MR images used in this study limits the motion model for being used in the clinical application such as radiotherapy planning. At this stage, our main focus is to demonstrate that GND-PCA can model the diaphragm motion with smaller number of sample data compared with regular PCA. The model cannot be applied for the clinical application due to the low MR image resolution. Higher resolution of MR image is required if one needs to apply the model for a clinical application.

Second is about shape modeling approach. In this study, we used simple spatial and temporal normalization. Although this simple normalization is sufficient to show the efficacy of GND-PCA compared with regular PCA, the use of other spatial normalization techniques such as active point distribution model [16] or nonrigid registration [17] such as thin-plate splines or cubic B-splines will provide a better diaphragm motion modeling. We will consider this issue in our future works.

Other consideration is that the manual segmentation of diaphragm area can affect the final results. Manual segmentation of diaphragm boundary is very subjective to the experience of the user. Hence, the variability of the diaphragm motion may change when the diaphragm area is resegmented. An automatic statistical shape model of diaphragm area from thoracic 2D MRI is needed to be developed for further work. Although the proposed method in [15] can be used to develop a statistical shape model of diaphragm, it is suited only for respiratory-gated CT data sets. Several adjustments need to be done to apply the method to thoracic 2D MRI.

## 5. Conclusion

We have developed a statistical method to model diaphragm motion using PCA. Time-sequential 2D MRI was constructed from a 4D MRI and extracted to obtain a 3D diaphragm motion model. Regular PCA and GND-PCA were then applied to construct model. In the experiment, we investigated that three eigenvectors or PCs with the largest eigenvalues are sufficient to accurately describe diaphragm motion model from ten healthy subjects. Model validation using leave-one-out showed that GND-PCA gives more stable reconstruction compared with regular PCA. This concludes that GND-PCA can model the motion better with a small number of sample data. Further works to be done include automatic segmentation of diaphragm area and investigation of compactness, generality, and specificity of the model.

## Figures and Tables

**Table 1:** Percent variations and cumulative contribution up to three principal components of 10 healthy subjects.

PC	%	Cum. %
1	97.4	97.4
2	1.1	98.5
3	0.7	99.2

**Table 2:** Comparison of the number of coefficients required to construct diaphragm motion model.

Regular PCA		GND-PCA	
PC	Coef.	Core tensor	Coef.
First PC	1	4 × 2 × 1	8
First two PCs	2	8 × 4 × 2	64
First three PCs	3	16 × 8 × 4	512

**Table 3:** Leave-one-out method validation using regular PCA: mean and average of maximum error position (in mm).

Used PC	Regular PCA		GND-PCA	
	Mean	Max	Mean	Max
1st PC	9.2	15.7	5.5	13.5
1st + 2nd PCs	6.5	17.3	2.4	8.1
1st–3rd PCs	6.3	17.2	1.3	5.5

## References

[1] von Siebenthal M, Székely G, Gamper U, Boesiger P, Lomax A, Cattin P (2007). 4D MR imaging of respiratory organ motion and its variability. *Physics in Medicine and Biology*.

[2] Tokuda J, Morikawa S, Haque HA (2008). Adaptive 4D MR imaging using navigator-based respiratory signal for MRI-guided therapy. *Magnetic Resonance in Medicine*.

[3] Masuda Y, Haneishi H 4D MR imaging of respiratory organ motion using intersection profile method.

[4] Werner R, Ehrhardt J, Frenzel T (2007). Motion artifact reducing reconstruction of 4D CT image data for the analysis of respiratory dynamics. *Methods of Information in Medicine*.

[5] Werner R, Ehrhardt J, Schmidt R, Handels H Modeling respiratory lung motion—a biophysical approach using finite element methods.

[6] McClelland JR, Blackall JM, Tarte S (2006). A continuous 4D motion model from multiple respiratory cycles for use in lung radiotherapy. *Medical Physics*.

[7] Georg M, Souvenir R, Hope A, Pless R Manifold learning for 4D CT reconstruction of the lung.

[8] Lyksborg M, Paulsen R, Brink C, Larsen R 4D lung reconstruction with phase optimization.

[9] Li R, Lewis JH, Jia X PCA-based lung motion model.

[10] Klinder T, Lorenz C, Ostermann J Free-breathing intra- and intersubject respiratory motion capturing, modeling, and prediction.

[11] King AP, Buerger  C, Schaeffter T (2010). Cardiac respiratory motion modelling by simultaneous registration and modelling from dynamic MRI images. *Biomedical Image Registration: 4th International Workshop*.

[12] Wein W, Cheng J, Khamene A Ultrasound based respiratory motion compensation in the abdomen.

[13] Xu R, Chen YW (2009). Generalized N-dimensional principal component analysis (GND-PCA) and its application on construction of statistical appearance models for medical volumes with fewer samples. *Neurocomputing*.

[14] McQuaid SJ, Lambrou T, Cunningham VJ, Bettinardi V, Gilardi MC, Hutton BF (2009). The application of a statistical shape model to diaphragm tracking in respiratory-gated cardiac PET images. *Proceedings of the IEEE*.

[15] Martin SJ, Dey J, King MA, Hutton BF Segmenting and tracking diaphragm and heart regions in gated-CT datasets as an aid to developing a predictive model for respiratory motion-correction.

[16] Cootes TF, Taylor CJ, Cooper DH, Graham J (1995). Active shape models—their training and application. *Computer Vision and Image Understanding*.

[17] Qiu Z, Tang H, Tian D Non-rigid medical image registration based on the thin-plate spline algorithm.

